# First experiences with the implementation of the European standard EN 62304 on medical device software for the quality assurance of a radiotherapy unit

**DOI:** 10.1186/1748-717X-9-79

**Published:** 2014-03-21

**Authors:** Angelika Höss, Christian Lampe, Ralf Panse, Benjamin Ackermann, Jakob Naumann, Oliver Jäkel

**Affiliations:** 1 Heidelberg Ion-Beam Therapy Center (HIT), Im Neuenheimer Feld 450, 69120 Heidelberg, Germany; 2 Department of Radiation Oncology, Heidelberg University Hospital, Im Neuenheimer Feld 400, 69120 Heidelberg, Germany; 3 Division of Medical Physics in Radiation Oncology, German Cancer Research Center (DKFZ), Im Neuenheimer Feld 280, 69120 Heidelberg, Germany

**Keywords:** EN 62304, Medical device software, Software life-cycle processes, EN ISO 14971, Risk management, Implementation, In-house manufacture, Radiotherapy

## Abstract

**Background:**

According to the latest amendment of the Medical Device Directive standalone software qualifies as a medical device when intended by the manufacturer to be used for medical purposes. In this context, the EN 62304 standard is applicable which defines the life-cycle requirements for the development and maintenance of medical device software. A pilot project was launched to acquire skills in implementing this standard in a hospital-based environment (in-house manufacture).

**Methods:**

The EN 62304 standard outlines minimum requirements for each stage of the software life-cycle, defines the activities and tasks to be performed and scales documentation and testing according to its criticality. The required processes were established for the pre-existent decision-support software FlashDumpComparator (FDC) used during the quality assurance of treatment-relevant beam parameters. As the EN 62304 standard implicates compliance with the EN ISO 14971 standard on the application of risk management to medical devices, a risk analysis was carried out to identify potential hazards and reduce the associated risks to acceptable levels.

**Results:**

The EN 62304 standard is difficult to implement without proper tools, thus open-source software was selected and integrated into a dedicated development platform. The control measures yielded by the risk analysis were independently implemented and verified, and a script-based test automation was retrofitted to reduce the associated test effort. After all documents facilitating the traceability of the specified requirements to the corresponding tests and of the control measures to the proof of execution were generated, the FDC was released as an accessory to the HIT facility.

**Conclusions:**

The implementation of the EN 62304 standard was time-consuming, and a learning curve had to be overcome during the first iterations of the associated processes, but many process descriptions and all software tools can be re-utilized in follow-up projects. It has been demonstrated that a standards-compliant development of small and medium-sized medical software can be carried out by a small team with limited resources in a clinical setting. This is of particular relevance as the upcoming revision of the Medical Device Directive is expected to harmonize and tighten the current legal requirements for all European in-house manufacturers.

## Background

**Figure 1:**
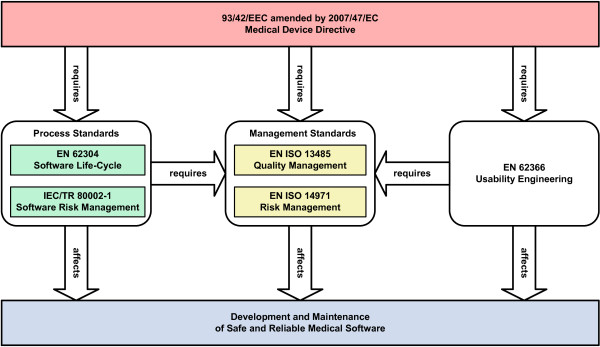
**Relationship of EN 62304 to other standards.** Compliance with the EN 62304 standard requires a quality management system, a risk management system and the application of usability engineering. Adherence to the applicable standards results in a presumption of conformity with the Medical Device Directive.

The European Council Directive 93/42/EEC [1] (Medical Device Directive) covers the placing on the market and putting into service of medical devices. Its Article 1 definition of a medical device includes the software necessary for its proper application, while its Annex IX points out that software, which drives or influences the use of a medical device, automatically falls in the same class as the device itself, both statements mainly referring to software embedded in electronic devices. Only the last amendment 2007/47/EC [2] of said directive clarifies the software-related requirements by an extension of the so-called essential requirements laid down in Annex I which are applicable to all medical devices regardless of whether they are placed on the market or not. It states that standalone software for diagnostic and/or therapeutic purposes is considered a medical device, and that for medical devices which incorporate or are software, the software must be validated according to the state-of-the-art taking into account the principles of development life-cycle, risk management, validation and verification. As the European state-of-the-art is represented by harmonized standards, this implies the application of the EN ISO 13485 standard [3] on quality management systems for medical devices, the EN 62304 standard [4] on medical device software life-cycle processes and the EN ISO 14971 standard [5] on the application of risk management to medical devices together with the IEC/TR 80002-1 technical report [6] providing guidance on its application to medical device software (Figure 1). Although medical software has been written and applied for at least three decades, the first edition of the EN 62304 standard harmonized in 2008 is the first standard dealing with standalone software (before, all software fell within the scope of the EN 60601-1-4 standard [7] on programmable electrical medical systems).

The Heidelberg Ion-Beam Therapy Center (HIT) [8-13] opened in November 2009 is the first hospital-based center in Europe where patients are treated with both protons and carbon ions in active beam application/raster scanning technique [14]. Two treatment rooms are equipped with a fixed horizontal beamline, while a third treatment room has a 360° rotational beam delivery system (gantry) which moves around the patient. As the Heidelberg University Hospital is not only the operator and user but also the manufacturer of the facility, it is in the position to advance this innovative and sophisticated technology instead of being confined to studying the efficacy of its clinical application at a fixed development stage. One of the core competencies required for this venture is the development of medical software compliant with state-of-the-art standards. Therefore, a pilot project was launched and carried out by a team of computer scientists, engineers and medical physicists aimed at the acquisition of the tools and techniques to efficiently implement the EN 62304 standard. The subject of this project was the development of a standalone decision-support software used during the quality assurance (QA) of treatment-relevant beam parameters. The software determines, on the basis of changes in the parameter settings of the device control units, which beam parameters were changed and subsequently suggests which QA procedure(s) should be carried out and passed before patient treatment is resumed after accelerator maintenance and/or beam adjustments. As it is intended to be used together with the HIT facility, and considered necessary for its proper application, it is a medical device in its own right to which the EN 62304 standard is applicable. In this paper we report on our first experiences with the implementation of said standard for this particular piece of in-house software.

## Methods

### General requirements of EN 62304

The EN 62304 standard outlines minimum requirements for each stage of the software life-cycle and defines the activities and tasks to be performed to provide adequate confidence that the software is safe and reliable. It introduces a risk-based approach – Class A through C where a failure of Class C software could result in death or serious injury – which ensures that medical device software including third party components, so-called Software of Unknown Provenance (SOUP), is subjected to a risk management correspondent to its hazard potential.

Compliance with the standard is achieved, if the predefined requirements for the applicable software safety class have been verifiably implemented, whereby there is a major difference in the requirements and thus in terms of time and costs between Class A and Class B code. Although the EN 62304 standard focuses on the software development process, which includes activities like development planning, requirements analysis, architectural design, unit implementation and verification, integration and integration testing, system testing and software release, no specific software development model like the V-Model or the Waterfall Model is requested.

The software maintenance and problem resolution processes are likewise important to enable the fast and efficient roll-out of software updates or patches and to address software errors detected after release. Another central demand of the standard is the traceability between software requirements including risk mitigations and system testing throughout the software and documentation while simultaneously providing a link between the different life-cycle phases. Well-integrated tools like a version control system, an issue tracking system and an automated build system can simplify and partially automate the implementation of substantial parts of the EN 62304 standard.

### Risk management according to EN ISO 14971

The safety and effectiveness of medical device software is dependent on the fulfillment of its intended use without causing unacceptable risks. The EN 62304 standard therefore requires compliance with the EN ISO 14971 standard which details the requirements for risk management to determine the safety of a medical device during the product life-cycle. Basically, the manufacturer has to establish an ongoing process to identify hazards, evaluate and control the associated risks and monitor the effectiveness of the controls. By evaluating the probability of the occurrence of harm and combining it with the severity of that harm, a measure of risk can be estimated. This value is compared to the manufacturer’s device-specific risk acceptance criteria, and if it is not in the acceptable range the risk needs to be mitigated by the implementation of one or more control measures which reduce the residual risk to an acceptable level.

The EN ISO 14971 standard defines a hierarchy of control measures, i.e. inherent safety by design should be preferred to protection measures in the device or its manufacture which in turn should be preferred to the provision of safety information to users. Objective evidence has to be provided that the selected control measures are implemented and effective, and it has to be ascertained that no known risks are increased and/or new risks are introduced by their implementation.

All documents required by the EN ISO 14971 standard are part of the risk management file the main purpose of which is to provide traceability for each identified hazard to the risk analysis, the risk evaluation, the implementation and verification of the control measures and the assessment of the acceptability of any residual risk(s). A preliminary endpoint of risk management is reached, if the overall residual risk is acceptable and an appropriate procedure is in place to obtain production and post-production information which has to be analyzed on a regular basis to determine if corrective or preventive action is required to fix a problem. If risks are greater than evaluated or new risks arise, the risk analysis has to be updated. Of course, this is also required if the medical device itself or its intended use is modified over the course of time.

### Purpose of the QA software under discussion

To deliver the pre-calculated therapeutic dose to the patient-specific target volume, all beam-influencing devices in the beamline of the HIT facility must be adjusted such that the requested ion type can be transported and accelerated from the source to the selected treatment room with the desired beam characteristics. Therefore, the accelerator control system (ACS) has to provide the control units of the participating devices with the required beam parameters. The valid beam parameter ranges are shown in Table 1.

**Table 1:** Beam parameters used for patient treatment

**Parameter**	**Steps**	**Ion type**	
		**Protons**	**Carbon ions**
Energy	255	48 – 221 MeV/u	88 – 430 MeV/u
Focus (FWHM)	1 (protons)/4 (carbon ions)	8 – 32 mm	6 – 12 mm
Intensity	9 (protons)/8 (carbon ions)	1·10^8^ – 3·10^10^ 1/s	1·10^7^ – 1·10^8^ 1/s
Gantry angle	360°	The gantry angle can be changed with a resolution of 0.01°; 36 steps are used for the interpolation of the settings	

**Figure 2:**
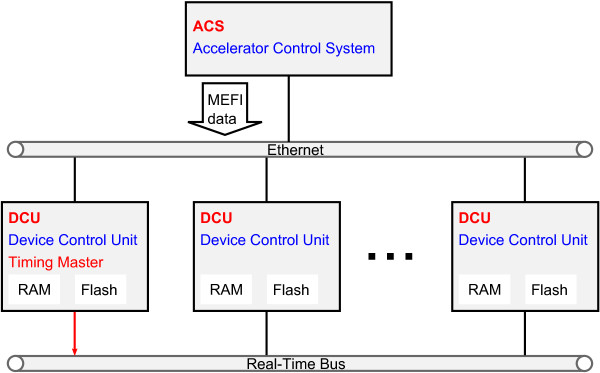
**ACS-DCU connectivity.** The MEFI data are sent from the ACS over an ethernet connection to the DCUs which are interconnected by a real-time bus system. An export of the MEFI data currently stored in the RAM or flash memory of all DCUs can be obtained in XML format.

The 177 device control units (DCUs) basically consist of network-enabled embedded controllers with real-time capabilities and both random access memory (RAM) and flash memory to store all device parameters for different combinations of **m**achine (= ion type), **e**nergy, **f**ocus and **i**ntensity (MEFI). While the device parameters for all MEFI combinations (MEFI data) for experimental modes are stored in volatile RAM which can be changed on the fly, the MEFI data validated for patient treatment are contained in the flash memory of each DCU respectively. Based on the required beam characteristics, which are represented by dedicated MEFI combinations, and the current operating mode the DCUs select the suitable device parameters. The ACS contains functionality which allows an export of the MEFI data currently stored in the RAM or flash memory of all DCUs in Extensible Markup Language (XML) format (Figure 2). The latter is referred to as “flashdump”. Currently, the size of each MEFI export file is about 500 MB. If an adjustment of beam parameters for patient treatment is required, e.g. for beam optimization after accelerator maintenance, it has to be applied and tested in the RAM first and then transferred (“flashed”) into the flash memory. The nature and extent of the subsequent validation of the flashed MEFI data depends on the modified devices and their respective beam influence.

To extract this information, a dedicated software called FlashDumpComparator (FDC) has been developed which not only compares the last and current flashdump, but also suggests based on the beam influence of the modified device(s) if and which QA procedure(s) should be carried out and passed in which treatment room(s) before clinical operation is resumed. These QA procedures include but are not limited to checks of the energy, focus, intensity and position of the beam. By using the FDC, the time required for QA is greatly reduced from approximately 48 hours (all beam parameters, all treatment rooms) to a few hours at the maximum (modified beam parameters, affected treatment rooms) so that beam adjustments and subsequent QA can be carried out without interruption of patient treatment. Due to its intended use and indirect impact on treatment quality through automated test selection the FDC was developed, tested and released according to the EN 62304 standard.

### Selection and integration of software tools

**Figure 3:**
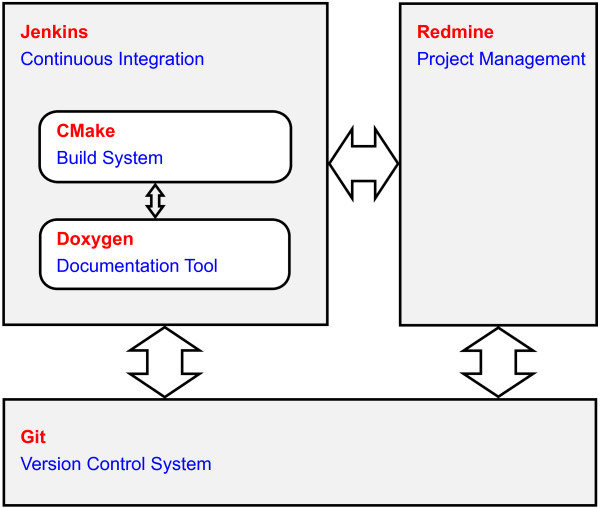
**Composition of utilized tools.** The code base is stored in Git (repository). Redmine is used to display and track differences of individual revisions of the source files, while Jenkins has access to Git to obtain the latest development state to automatically start build processes and extract documentation.

The requirements of the EN 62304 standard regarding the software maintenance and problem resolution processes are difficult to implement without proper tools, although this is not imperative. Therefore, a considerable amount of time was spent on the selection and integration of compatible state-of-the-art tools supporting those processes. Figure 3 shows the composition of the utilized tools. They are freely available and have interfaces which reduce the dependency on their long-term availability. As these software tools are not part of the FDC the level of concern is generally low, nevertheless all used functionality was independently validated (i.e. by a computer scientist separate from the development team).

**Redmine** is a role-based project management system which can be used for issue tracking, forums, wikis, repository browser, news and file management. It can handle multiple projects and is excellently scalable. Its features are modules which can be enabled or disabled for each project, and it has an interface to add functionalities by plugins. **Jenkins** is a web-based system which provides continuous integration for software development. It is expandable by a large number of plugins which can be used to adapt it to the own requirements for a suitable build process. **Git** is used as version control system and contains the source code and other files required during the build process. It belongs to the group of decentralized version control tools which means that each user has a copy of the complete repository with the entire history. Jenkins requires access to the Git repositories to obtain the latest development state to automatically start build processes. The repository browser of Redmine uses Git to display and track differences of individual revisions of the source files.

CMake and Doxygen are supporting tools which are used by Jenkins before and after the build process. **CMake** is a cross-platform build system to control the software builds by platform-independent script files. With these script files, native make files and projects for integrated development environments like Microsoft Visual Studio can be generated. **Doxygen** extracts documentation from source files comments which are present in a particular syntax. The produced documentation can be provided as HyperText Markup Language (HTML) or Portable Document Format (PDF).

## Results

### Risk analysis

A rapid prototyped version of the FDC written in C++ was used as the basis for the requirements analysis by an interdisciplinary 6-person team of accelerator physicists, computer scientists, medical physicists and software testers which was subsequently turned into a requirements specification. All requirements were itemized in tabular form with unique IDs to facilitate the identifiability and traceability of each requirement, and the complete requirements specification was subjected to an informal review against a checklist to ascertain comprehensibility, verifiability and consistency.

Subsequently, a risk analysis was carried out to determine, if risks above the acceptance level arose from the specified requirements and/or if any additional requirements resulted from the necessity to mitigate risks. The definition of the risk acceptance criteria (categories for severity and occurrence) was based on the assumption that 100 patients are treated per day in a time interval of 25 years and the FDC is applied 10 times per year at the maximum. The underlying worst case scenario is a false negative result of the FDC, i.e. the indication on the FDC result print that no QA is required although at least one beam parameter is out of tolerance which catastrophically (= death or serious injury) affects the treatment of all subsequent patients. The examples of software causes listed in Annex B of the IEC/TR 80002-1 report were used to generate a list of hazards which were considered during the risk assessments to achieve completeness.

**Figure 4:**
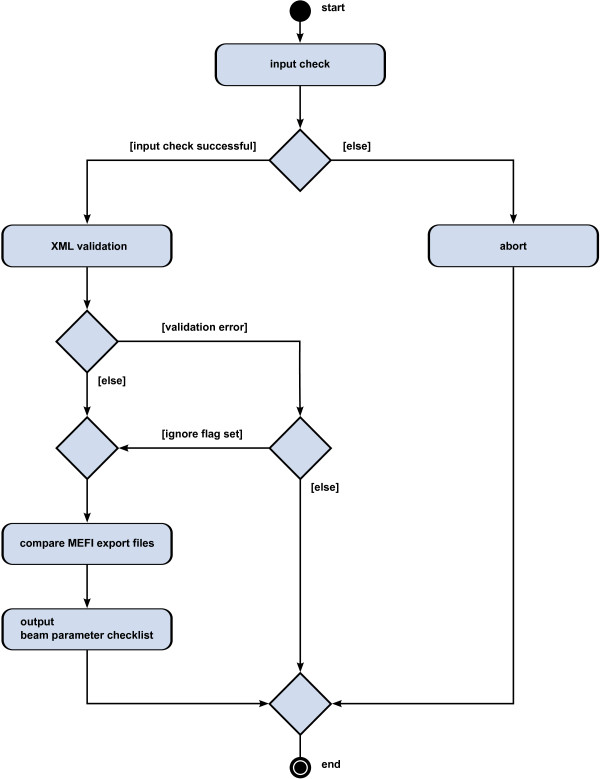
**UML representation of FDC workflow.** If the input parameters and MEFI export files are processable, an XML validation is performed which can be skipped for research purposes. If this validation is successful, the last and current flashdump are compared and a beam parameter checklist including the detected differences is stored to a file.

The functions of the FDC discussed in the risk analysis – one after another for each phase of the software life-cycle – were derived from an activity diagram (Unified Modelling Language (UML) representation) of the FDC workflow. Figure 4 shows a simplified version of this activity diagram. First, there is a check if the input parameters and files are processable. Then, an XML validation is performed to check the MEFI export files against a predefined XSD (XML Schema Definition) file. If this validation is successful, the last and current flashdump are compared and a beam parameter checklist including the detected differences is stored to a file. In case of an error, the program execution is terminated. If the ignore flag is set, the FDC continues in spite of validation errors, but only the detected differences and no beam parameter checklist are printed. In total, the time required for risk assessments prior to the first release of the FDC was 87.25 person hours.

### Risk mitigations

In the risk analysis error causes like wrong startup flags, knowledge base and output filters, contentswise or syntactically wrong flashdumps, dysfunctional SOUP (a Windows Server 2008 and the XML parser library Xerces), the impact of the operational environment and errors introduced during release and maintenance were addressed. The highest initial risk scores were attributed to the comparison of identical or outdated flashdumps, programming errors and incorrect entries of device properties in the knowledge base.

The counteracting **system design mitigations** included the addition of metadata, timestamps and checksums to the flashdumps, their XSD validation, the recording of each flash procedure by the ACS, a matching of DCU entries in flashdumps and knowledge base, the abortion of the FDC on errors and some functionalities of the tools provided by the integration server (e.g. revision control, automated builds, regression tests, static code analysis). The **safety information mitigations** consisted of procedural and operating instructions. Of course, system testing against the requirements specification (i.e. 63 test cases covering the range of functions and the software performance) and acceptance testing by a medical physics expert were also required **quality assurance mitigations** as was usability testing against the instructions for use. The latter confirmed that a dedicated FDC user training was not required as a risk mitigation.

Besides, a software maintenance and problem resolution process was established which also allows for changes of the FDC software and/or beam-influencing devices. In total, 38 control measures were implemented according to the four-eye-principle, and their effectiveness was additionally verified by an external auditor. For the first release of the FDC, the time required for the realization of the risk mitigations – which included a considerable amount of software reengineering and verification/validation – added up to 240 person days, while the sample audits of the corresponding objective evidence took 26.5 person hours only. Provided that all those control measures are fully implemented and long-term effective the overall residual risk when using the FDC is acceptable.

### Software development

The actual software development started with the outlining of development guidelines and the definition of a procedure model. Essentially, it is based on the V-Model except for some iterative portions which result from the consideration of other standards (e.g. the EN 62366 [15] standard on the application of usability engineering to medical devices) and the chosen integration technique. We opted for the V-model because the activities and their dependencies according to the EN 62304 standard can easily be mapped to it. Regarding software configuration management basic provisions were established concerning version control, usage of repositories, dealing with SOUP and change control. A tripartite software version number was specified which is automatically incorporated in the source code during the software build process. By assigning a revision number to incrementally different versions the software can be tracked to a unique development state which facilitates the locating and fixing of bugs. The guideline regarding the usage of repositories with the version control system Git defines which classes of files may be stored in which directories of the repository. The repository contains all source code and other files required to create the software and perform automatic tests. The SOUP integrated in the FDC is referenced as well in order to document automatically and comprehensively which version of SOUP is contained in which version of the FDC.

From a risk management perspective it is also required to review and assess the error lists of SOUP manufacturers on a regular basis to mitigate SOUP-induced risks as may be necessary. The software maintenance and problem resolution process ensures that proposed changes to the FDC (bug fixes or functional expansions) are implemented in a coordinated manner which includes a review and approval of each change, possibly an update of the risk analysis and/or the documentation (requirements specification, test specification, user manual etc.), a suitable amount of regression testing and the release of a new version.

### Software context and testing

Large parts of the software architecture were adopted from the initial FDC prototype. As the FDC is not only the subject of a risk analysis, but also the basis for a risk mitigation (verification of newly flashed MEFI data prior to the resumption of clinical operation) often used within the risk analysis of the HIT facility, the software safety class B of the FDC was derived from the risk scores applied in the superordinate risk analysis. A decomposition of the FDC into several software items, which could have been classified separately, was not carried out as no benefit was expected due to the size of the FDC (about 8400 lines of code) and the chosen software architecture.

While UML was used for the visualization of the software architecture, the documentation of the interfaces among the software units was realized by using source code embedded comments which were extracted during the build process to create an Application Programming Interface (API) document in PDF. Due to the continuous integration services provided by Jenkins the software build process is automated. Besides, a static code analysis, coding style checks and regression tests are carried out during each build, and their results are summarized on the dashboard of Jenkins.

A new version of the FDC may only be released if there are no unchecked errors resulting from the static code analysis. Due to the **regression tests** the crucial FDC functions are automatically tested after each code change. A higher level of testing is realized by the **system test** which includes test cases covering all functional and non-functional requirements as well as negative test cases (e.g. using invalid input). The system test documentation includes but is not limited to the test case specification – test cases and procedures and their links to the requirements specification via a unique ID – and the respective test results. A priority (1-3) is assigned to each test case, and it is defined in the system test documentation which tests with which priority need to be passed for a major and minor FDC release. **Usability testing** was carried out in the form of participatory observation meaning that an unbiased person was required to perform a set of predefined tasks with the help of the instructions for use only. As a consequence, many options (startup flags) were dropped, conflated and renamed to increase the usability of the FDC.

### Requirements traceability

One of the objectives of the pilot project was to work paperless whenever this was reasonable and feasible. Therefore, the issue tracking system of Redmine is used by the software maintenance and problem resolution process and the risk management process. The information about a change request or risk mitigation is stored in a ticket. Different types of tickets, so-called trackers, were defined according to their objectives, e.g. error, new feature, improvement, task (code-independent) and risk mitigation. A ticket is created via a tracker-dependent input mask which requires information like subject, description, priority, target version and status. Each ticket has an automatically generated unique ID and is assigned to a user who is responsible for the proper resolution of the issue that it contains. The ticket ID can be quoted when the associated code change is checked into the repository of Git thus creating a direct link between the requirement and its implementation. Code-independent tickets can be closed by attaching meaningful documents like operating procedures, test results or the instructions for use.

The processing stages of each ticket are recorded in its history which is particularly relevant for tickets which are handled by multiple users according to definition. Especially the state transitions defined by the risk management process, where a mitigation is handled by four different persons each of which can reject the specified requirement or chosen implementation (e.g. because the requirement is not practical or the implementation is not target-aimed), were modelled in the ticket life-cycle. This means that at any given stage only the assigned user can process such a ticket which concludes with a change of state (the selectable states are dependent on the current user role) and a handover to another user. The endpoint is the closure of the ticket by the risk manager after the successfully audited ticket has been presented to and accepted by the risk analysis team by which the risk mitigation was specified.

The prerequisites for the release of a new FDC version as an accessory to the HIT facility are that the predefined set of test cases and all associated risk mitigation tickets have been concluded successfully, and that the software documentation including the test summary report and the instructions for use is up-to-date and complete. Besides, the software package and associated documentation generated during the build process must be archived before the new FDC version is rolled out to the target computers.

## Discussion

### Experience with the implementation of EN 62304

Standalone software qualifies as a medical device, if it performs actions for the benefit of individual patients and is used for a medical purpose or drives, monitors or influences the performance or use of a medical device. Although the application of standards is voluntary, compliance with the EN 62304 standard provides a presumption of conformity with some of the essential requirements for medical devices laid down in Annex I of the European Council Directive 93/42/EEC. However, a whole range of other standards and requirements needs to be considered before medical software may be placed on the market or put into service. If an applicable standard or part of it is not observed, the manufacturer has to provide objective evidence that equivalent safety has been achieved by alternate means. Therefore, it is advisable and mostly also easier to adhere to the standard as closely as possible, and to state the reasons for deviations and/or omissions. The definition of the activities and the creation of the documents required by the EN 62304 standard are time-consuming at first, but if they are drafted sufficiently generic many of them can be adopted as they stand to follow-up projects. The same applies to the careful selection, implementation and maximum possible integration of the aforementioned software tools. The existence of a well-elaborated prototype of the FDC at project launch has demonstrated the importance of a timely preparation of a requirements specification and subsequent risk analysis as even the developers of the prototype stated deviating requirements and proposed a variety of changes when asked to set the former down in writing and take a safety-related perspective. This resulted in a massive amount of unforeseen changes – simplifications and expansions, most of them originating from the risk analysis – which substantially delayed the project and could have been avoided if considered at an earlier date. Beyond that, the implementation of the traceability requirements proved to be nontrivial as several attempts were required to find an approach which reduced the expenditure for the adaptation of our 11 requirements documents transposing the EN 62304 standard to an acceptable level in case of software changes.

### Software adaptation after initial roll-out

Contrary to expectations, the FDC had to be updated only months after its first release due to a potential hazard which was not considered in the initial risk analysis but became apparent during routine use, namely the possibility to compare MEFI export files from flash memory and RAM rather than flash memory exports from subsequent points in time. To keep the functionality desired for accelerator adjustments, but eliminate it as an error source for patient treatment, a tag indicating the “dump type” was added to the flashdumps which is checked by the FDC. The corresponding risk mitigation requires the FDC to only compare flash memory exports by default. If a comparison of MEFI export files from flash memory and RAM is required, the user has to add a dedicated startup flag and this deviation from the intended use is clearly indicated on the result print. The experiences gained during this minor change of the FDC were used to streamline the software maintenance and problem resolution process with the objective to shorten the update-cycle to a few days for minor and a few weeks for major FDC releases. Especially the need of test automation became apparent. For the first FDC release, it took 5 days to create the test data for 42 test cases and carry them out manually. After script-based test automation, which also included the automatic generation of test data, only 6 hours were required to complete 63 test cases prior to the second major FDC release.

### Revision of the European Council Directive 93/42/EEC

At present, a fundamental revision of the regulatory framework for medical devices is under way based on a proposal for a regulation of the European Commission in September 2012 [16] which has been adopted by the European Parliament in October 2013 (partial vote at first reading, 347 voted-in amendments) [17] and transferred back to the responsible Environment, Public Health and Food Safety Committee. Its aim is to overcome the substantial divergences in the interpretation and application of the existing legislation and to close the regulatory gaps which exist with regards to certain products. Unlike a directive which sets out a goal that all European countries must achieve by transposition into national law, a regulation becomes immediately enforceable as law in all member states simultaneously thus creating a level playing field for manufacturers, notified bodies and competent authorities. The current directive leaves the decision to which extent in-house manufacturing is subjected to legal requirements to the national legislator, so there is a maximum range of national law from exemption (e.g. United Kingdom) to CE marking (e.g. Austria). In Germany, the conformity assessment procedure is simplified, but all applicable essential requirements of Annex I must be met. The future regulation considers in-house manufactured devices as being put into service thus subjecting them to the law but exempts them from CE marking, provided that their manufacture and use occur under a quality management system. While it is beyond the scope of this paper to discuss all software-related changes, some alterations of its scope and definitions are noteworthy as they broaden the range of products falling under the law considerably like the introduction of indirect medical purposes, the inclusion of the prediction of disease and the expansion of the accessory definition.

## Conclusions

The pilot project carried out at the HIT facility clearly demonstrates that the interpretation and implementation of the EN 62304 standard, especially the selection and integration of appropriate software tools and the test automation by means of dedicated in-house software, is not feasible without appropriately qualified staff. Nevertheless, it has also been shown that a standards-compliant development of small and medium-sized medical software can be carried out by a small team with limited resources although the initial effort is significant and a learning curve must be overcome. We would like to emphasize that the acquisition of knowledge on the requirements and implementation of the EN 62304 standard is gaining importance as a revision of the European regulatory framework for medical devices is under way which will have an impact on all European in-house manufacturers because it tightens the legal requirements and rescinds all national provisions which have obviated or simplified compliance so far. Apart from the unclear concept of indirect medical purposes, the major expansion of the accessory definition is highly questionable. If implemented as proposed, any software which **assists** the medical functionality of a medical device in view of its intended purpose (now: is intended to be used together with a medical device to **enable** it to be used in accordance with its intended purpose) is an accessory and therefore regulated as a medical device. As even macros, scripts, dynamically linked libraries and batch files can be standalone software in the meaning of the MEDDEV 2.1/6 guidance document [18] on the qualification and classification of standalone software this might open Pandora’s box for many radiation oncology and medical physics departments all over Europe.

## Abbreviations

ACS: Accelerator control system; API: Application programming interface; DCU: Device control unit; EC: European community; EEC: European economic community; EN: European standard; FDC: FlashDumpComparator; FWHM: Full width at half maximum; HIT: Heidelberg ion-beam therapy center; HTML: HyperText markup language; ID: Identifier; IEC: International electrotechnical commission; ISO: International organization for standardization; MB: Megabyte; MEDDEV: MEDical DEVices guidance document; MEFI: Machine energy focus intensity; PDF: Portable document format; QA: Quality assurance; RAM: Random access memory; SOUP: Software of unknown provenance; TR: Technical report; UML: Unified modelling language; XML: Extensible markup language; XSD: XML schema definition.

## Competing interests

The authors declare that they have no competing interests.

## Authors’ contributions

RP wrote the initial FDC prototype and expanded it together with CL according to the requirements specification of BA and OJ. CL selected and integrated the described software tools, besides he and RP designed and implemented the tools for test automation. All authors participated in the execution of the risk analysis and implementation of the risk mitigations, both overseen by AH. Acceptance testing was carried out by BA. All authors read and approved the final manuscript.
